# Avian WNT4 in the Female Reproductive Tracts: Potential Role of Oviduct Development and Ovarian Carcinogenesis

**DOI:** 10.1371/journal.pone.0065935

**Published:** 2013-07-02

**Authors:** Chul-Hong Lim, Whasun Lim, Wooyoung Jeong, Jin-Young Lee, Seung-Min Bae, Jinyoung Kim, Jae Yong Han, Fuller W. Bazer, Gwonhwa Song

**Affiliations:** 1 WCU Biomodultion Major, Department of Agricultural Biotechnology, Seoul National University, Seoul, Republic of Korea; 2 Department of Animal Resources Science, Dankook University, Cheonan, Republic of Korea; 3 Center for Animal Biotechnology and Genomics and Department of Animal Science, Texas A&M University, College Station, Texas, United States of America; 4 Division of Biotechnology, College of Life Science and Biotechnology, Korea University, Seoul, Republic of Korea; Institut Jacques Monod, France

## Abstract

The wingless-type MMTV integration site family of proteins (WNTs) is highly conserved secreted lipid-modified signaling molecules that play a variety of pivotal roles in developmental events such as embryogenesis, tissue homeostasis and cell polarity. Although, of these proteins, WNT4 is known to be involved in genital development in fetuses of mammalian species, its role is unknown in avian species. Therefore, in this study, we investigated expression profiles, as well as hormonal and post-transcriptional regulation of WNT4 expression in the reproductive tract of female chickens. Results of this study demonstrated that WNT4 is most abundant in the stromal and luminal epithelial cells of the isthmus and shell gland of the oviduct, respectively. WNT4 is also most abundant in the glandular epithelium of the shell gland of the oviduct of laying hens at 3 h post-ovulation during the laying cycle. In addition, treatment of young chicks with diethylstilbestrol (DES, a synthetic estrogen agonist) stimulated WNT4 only in the glandular epithelial cells of the isthmus and shell gland of the oviduct. Moreover, results of our study demonstrated that *miR-1786* influences WNT4 expression via specific binding sites in its 3′-UTR. On the other hand, our results also indicate that WNT4 is expressed predominantly in the glandular epithelium of cancerous ovaries, but not in normal ovaries of hens. Collectively, these results indicate cell-specific expression of WNT4 in the reproductive tract of chickens and that it likely has crucial roles in development and function of oviduct as well as initiation of ovarian carcinogenesis in laying hens.

## Introduction

The chicken oviduct is well known as a model for research on hormone action, including effects of estrogen and progesterone [1]. Especially, estrogen is known as the hormone responsible for growth of the yolk and follicle, and the process of calcium metabolism for formation of the egg shell and the process of oviposition or laying of the egg [1]. In addition, estrogen has also a crucial role in the process of the synthesis of egg white proteins in the oviduct [2]. Furthermore, formation of the tubular glands and differentiation of the epithelial cells including goblet and ciliated cells in the chicken oviduct are induced by estrogen [3].

WNT4 is a secretory signaling protein concerned with multiple processes in organ development including formation of kidney, mammary gland and adrenal gland, as well as sexual development in mammals [4]. Of particular note, WNT4 is a key player in the development and differentiation of the female reproductive system. In mice, the Wnt4 signaling pathway participates in folliculogenesis, luteogenesis and steroidogenesis of granulosa cells and in the regulatory processes of stromal cell proliferation and differentiation for survival and development of embryos within the uterine lumen [5]. Interestingly, several genes involved in the WNT signal pathway(s) are regulated by estrogen. In fact, the WNT4-FZD2 signaling pathway is activated by binding of estrogen to estrogen receptor alpha (ESR1) in the uterus [6] and in somatotrophs that produce growth hormone in the anterior pituitary gland of rodents [4]. Furthermore, over-expression of WNT4 leads to the development of malignant tumors. Indeed, the elevated expression of WNT4 is frequently observed in many breast cancer patients which implies that it's aberrant expression leads to abnormal cell proliferation and breast cancer in women [7].

There is little known about the expression or function of WNT4 in the reproductive tract of female chickens. Therefore, the objectives of this study were to: 1) reveal cell-specific expression patterns of WNT4 in the chicken oviduct during the reproductive cycle; 2) determine whether estrogen regulates expression of WNT4 during development of the chick oviduct; 3) determine whether WNT4 expression is mediated through post-transcriptional activity of specific microRNAs and 4) compare the expression pattern of WNT4 between normal and cancerous ovaries. Results of the present study provide novel insights into the *WNT4* gene of chickens with respect to cell-specific expression and hormonal regulation of its expression during oviduct development, the laying cycle and development of ovarian carcinogenesis in laying hens.

## Results

### Expression and localization of *WNT4* in the chicken oviduct

**Figure 1:**
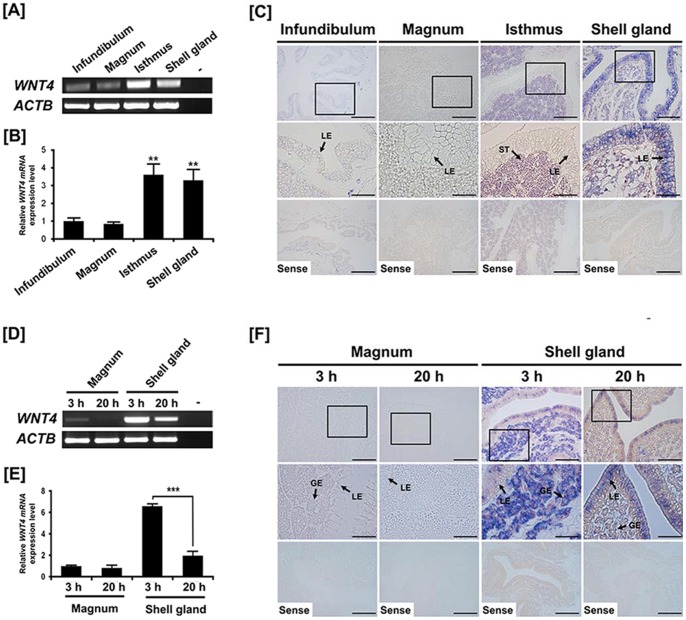
Expression and localization of *WNT4* in the chicken oviduct. Both RT-PCR [A] and quantitative PCR [B] analyses were performed using cDNA templates from each segment of the chicken oviduct. These experiments were conducted in triplicate and normalized to control *ACTB* expression. [C] *In situ* hybridization analysis for cell-specific changes in expression of *WNT4* in the each segment of the chicken oviduct. Both RT-PCR [D] and quantitative PCR [E] analyses were performed using cDNA templates from the magnum and the shell gland segment at 3 h and 20 h after ovulation. [F] *In situ* hybridization analysis for cell-specific changes in expression of *WNT4* in the magnum and the shell gland at 3 h and 20 h after ovulation. Legend: ST, stromal cells; GE, glandular epithelium; LE, luminal epithelium. *Scale bar* represents 50 μm (the first and the third horizontal panels) and 20 μm (the second horizontal panel). The tissue samples were from oviducts of 1- to 2- year-old female laying hens (n = 5). The asterisks denote statistically significant differences (***P<0.001 and **P<0.01).

Anatomically, the chicken oviduct consists of four segments: the infundibulum (site of fertilization), magnum (production of components of egg-white), isthmus (formation of the shell membrane) and shell gland (formation of the egg shell). Results of RT-PCR analysis indicated a high level of *WNT4* mRNA expression in the isthmus and the shell gland as compared with the infundibulum and the magnum (Figure 1A). Further, quantitative PCR analysis revealed that *WNT4* mRNA levels in the isthmus and the shell gland were 3.59- and 3.29-fold (*P*<0.01) greater, respectively, than for the infundibulum, and its expression decreased 0.16-fold in the magnum (Figure 1B). To determine localization of *WNT4* mRNA in the chicken oviduct, *in situ* hybridization analysis was performed (Figure 1C). The *WNT4* mRNA was most abundant in stromal cells and luminal epithelia (LE) of the isthmus and the shell gland, respectively. However, little or no mRNA was detected in the infundibulum and the magnum of the chick oviduct.

### Expression and localization of *WNT4* in the chicken oviduct at different stages of the laying cycle

We previous reported spatial and temporal changes in gene expression in the oviduct of laying hens at different stages of the laying cycle [8]. In order to detect cell-specific localization of *WNT4* mRNA in the chicken oviduct between 3 h and 20 h after ovulation, RT-PCR, quantitative PCR and *in situ* hybridization analyses were performed. As illustrated in Figure 1D, RT-PCR analysis detected the highest level of *WNT4* mRNA expression at 3 h post-ovulation in the shell gland and lowest expression at 20 h post-ovulation in the shell gland, but little or no detectable *WNT4* mRNA in the magnum at either time point. In addition, quantitative PCR analysis revealed that expression of *WNT4* mRNA was 3.32-fold (*P*<0.001) at 3 h than at 20 h post-ovulation in the shell gland, but changes in expression of *WNT4* mRNA were not different between 3 h and 20 h post-ovulation in the magnum (Figure 1E). Consistent with these results, *in situ* hybridization analyses indicated that *WNT4* mRNA was predominantly localized to the glandular epithelium (GE) of the shell gland at 3 h post-ovulation and it was also detected to a lesser extent in LE of the shell gland at both time points (Figure 1F). However, there is either no or very little expression of *WNT4* in the magnum.

### Effects of DES on *WNT4* expression in the chicken oviduct

**Figure 2:**
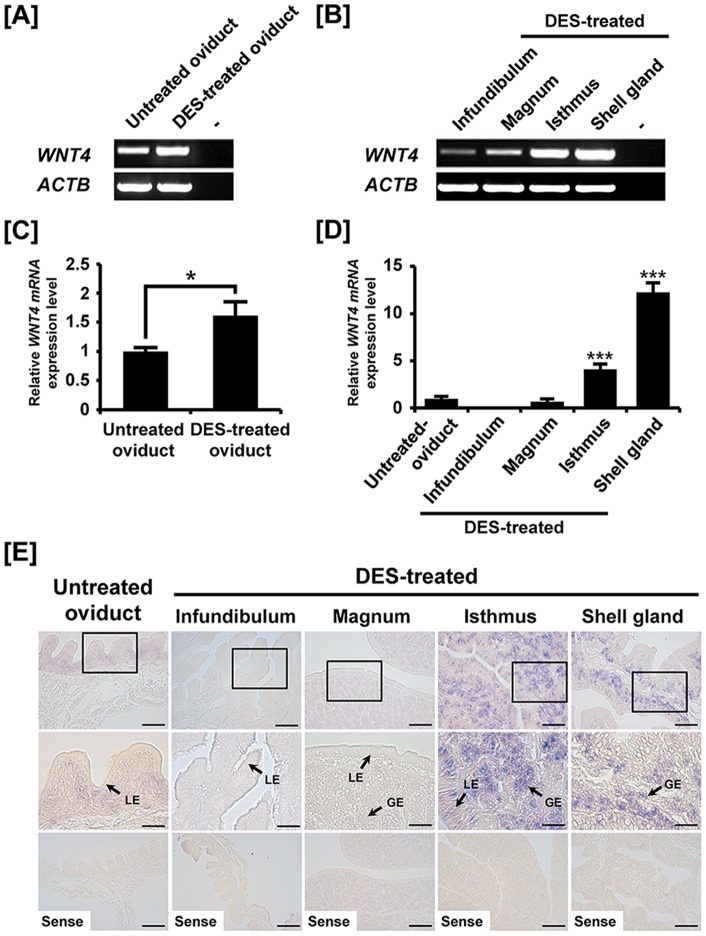
Effect of DES on tissue-specific expression of chicken *WNT4*. Both RT-PCR [A and B] and quantitative PCR [C and D] analyses were performed using cDNA templates from DES-treated and untreated oviducts. These experiments were conducted in triplicate and normalized to control *ACTB* expression. [E] *In situ* hybridization analyses revealed cell-specific expression of *WNT4* mRNA in oviducts of DES-treated and untreated chicks. Cross-sections of the four segments of chicken oviduct (infundibulum, magnum, isthmus, and shell gland) from chicks treated with DES or vehicle were hybridized with antisense or sense chicken *WNT4* cRNA probes. Legend: GE, glandular epithelium LE, luminal epithelium. *Scale bar* represents 50 μm (the first and the third horizontal panel) and 20 μm (the second horizontal panel). The tissue samples were used from 37-day-old chick oviducts (n = 5). The asterisks denote statistically significant differences (***P<0.001 and *P<0.05).

Cell-specific expression of *WNT4* mRNA in the oviduct of mature hens suggested regulation by estrogen during development of the chicken oviduct. Because diethylstilbestrol (DES) is a synthetic estrogen that binds to estrogen receptors with similar effect of the natural estrogen, 17β-estradiol [1], [9], [10], we determined effects of DES and reported that DES regulates growth, development and cytodifferentiation of the immature chick oviduct [9]. Likewise, we examined the effects of DES on expression of *WNT4* mRNA in the chicken oviduct in the present study. As illustrated in Figure 2A and 2B, expression of *WNT4* mRNA increased in DES-treated oviducts as compared with untreated oviducts. Further, quantitative PCR analysis confirmed that *WNT4* expression increased 1.6-fold (*P*<0.05) in DES-treated as compared to control oviducts (Figure 2C). In addition, DES treatment stimulated 4.1- and 12.3-fold increases (*P*<0.001) in *WNT4* mRNA in the isthmus and the shell gland, respectively (Figure 2D). To determine localization of *WNT4* mRNA in chick oviducts treated with DES, *in situ* hybridization analysis was used to reveal that *WNT4* mRNA is expressed predominantly expressed in GE of the isthmus and the shell gland (Figure 2E). There was little or no detectable *WNT4* mRNA in the infundibulum and magnum.

### Post-transcriptional regulation of microRNA affecting *WNT4*

**Figure 3:**
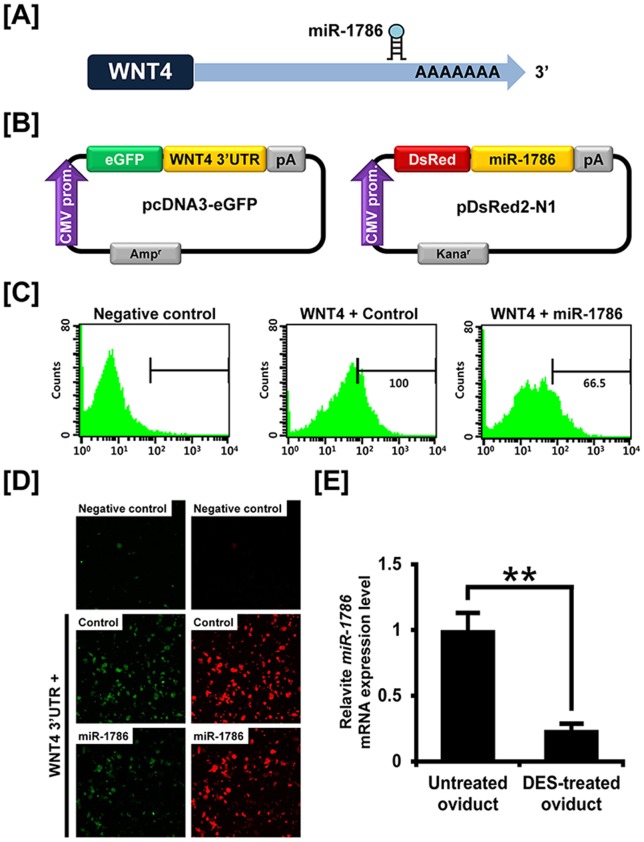
*In vitro* target assay of *miR-1786* on *WNT4* transcript. [A] Diagram of *miR-1786* binding site in the 3′-UTR *of the WNT4* gene. [B] Expression vector maps for eGFP with the 3′-UTR of *WNT4* and Ds-Red with *miR-1786*. The 3′-UTR of the *WNT4* transcript was subcloned between the eGFP gene and the polyA tail to generate the fusion construct of the GFP transcript following the miRNA target 3′-UTR (pcDNA-eGFP-3′UTR) (upper panel) and miRNA expression vector was designed to co-express DsRed and miRNA (pcDNA-DsRed-miRNA) (lower panel). [C and D] After co-transfection of pcDNA-eGFP-3′UTR for the *WNT4* transcript and pcDNA-DsRed-miRNA for the *miR-1786*, the fluorescence signals of GFP and DsRed were detected using FACS [C] and fluorescent microscopy [D]. [E] Quantitative expression of miR-1786 between untreated and DES-treated oviducts of chicks was analyzed by qRT-PCR. This experiment was normalized to control U6 snRNA expression. The asterisk denotes statistically significant differences (**P<0.01).

To demonstrate the possibility that expression of WNT4 is affected through the post-transcriptional regulation by miRNAs, we performed a miRNA target validation assay. We identified potential miRNA binding sites within the 3′-UTR of the *WNT4* gene using the miRNA target prediction database (miRDB; http://mirdb.org/miRDB/) which revealed only one putative binding site for *miR-1786*. Therefore, we determined whether *miR-1786* influenced expression of the *WNT4* gene via its 3′-UTR. As illustrated in Figures 3C and 3D, the expression level of GFP-expressing cells decreased 33.5% (*P*<0.05) in the presence of *miR-1786*, as compared with control values based on FACS and fluorescence microscopy analyses. In addition, *miR-1786* expression was reduced 75% (*P*<0.01) in the DES-treated oviducts as compared to untreated oviducts of chicks through miRNA-specific quantitative RT-PCR analysis (Figure 3E). These results reveal that *miR-1786* regulates WNT4 expression post-transcriptionally *in vivo* by binding directly to the WNT4 transcript.

### Differential expression of *WNT4* between normal and cancerous ovaries of hens

**Figure 4:**
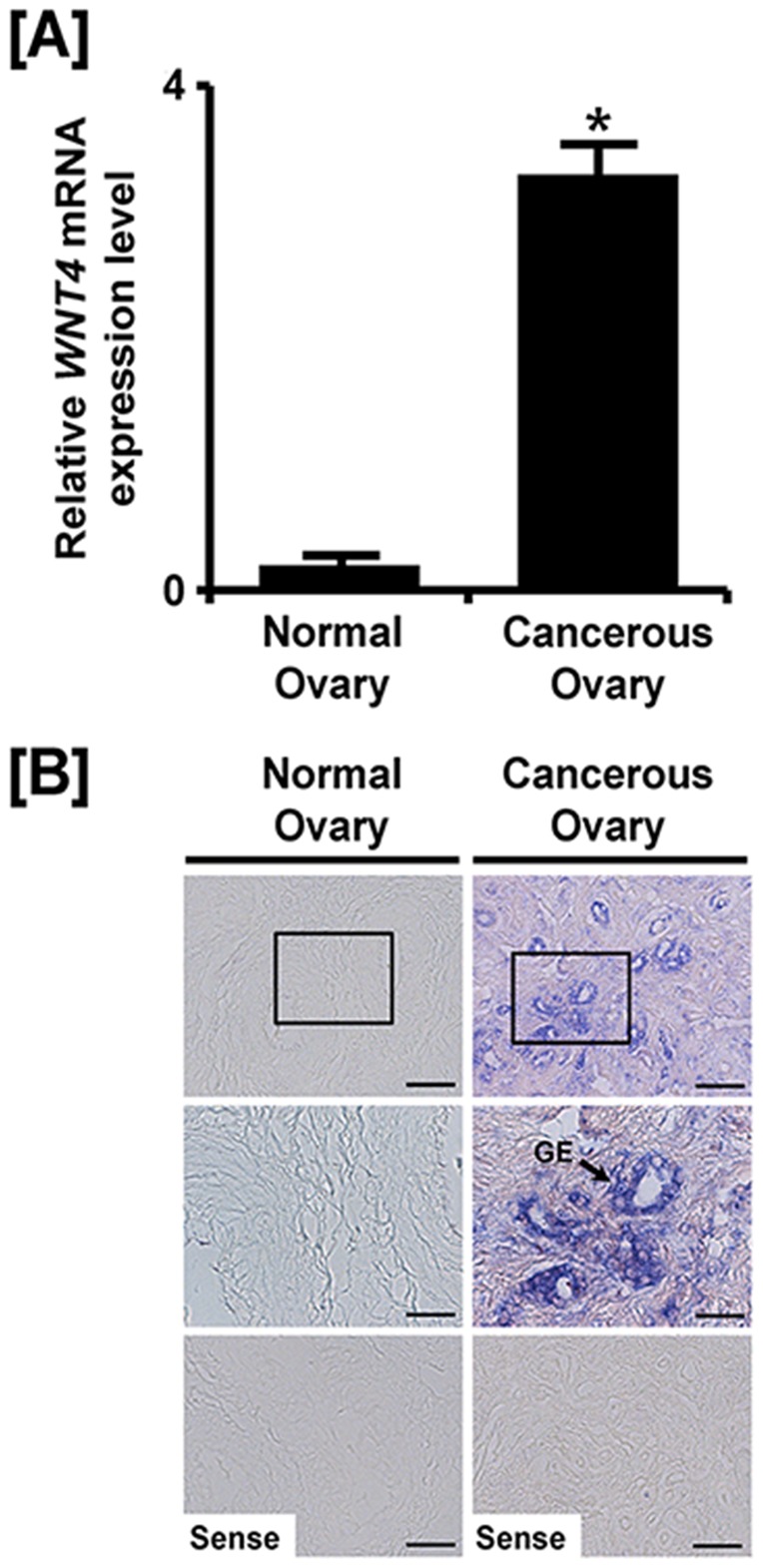
Expression and localization of *WNT4* between normal and cancerous ovaries. [A] Quantitative PCR analysis was performed using cDNA templates from normal and cancerous ovaries. [B] *In situ* hybridization analyses revealed cell-specific expression of *WNT4* mRNA between normal and cancerous ovary. Legend: GE, glandular epithelium. *Scale bar* represents 50 μm (the first and the third horizontal panel) and 20 μm (the second horizontal panel). The tissue samples were from normal and cancerous ovaires of 2- to 3- year-old laying hens (n = 5). The asterisks denote statistically significant differences (*P<0.05).

The laying hen is a unique animal model for study of human epithelia-derived ovarian cancer research. This is because they spontaneously develop ovarian cancer of the surface epithelium of the ovaries at a high rate and are useful for development of biomarkers for detection and early diagnosis of ovarian cancer, as well as for discovery of anti-cancer drugs/biomaterials [11]. There is evidence that epithelial cell-derived ovarian cancer (EOC) in women may originate from epithelial cells of the oviduct [12], [13]. Likewise, in chickens, Trevino *et al* [14] reported that about 50% of up-regulated genes in EOC of laying hens are oviduct-associated genes. In addition, we reported that several estrogen-stimulated genes, including *serpin peptidase inhibitor, clade B, member 11* (*SERPINB11*) [15], *SERPINB3* [16], *cathepsin B* (*CTSB*) [17], *S-adenosylhomocysteine hydrolase-like protein 1 (AHCYL1)* [18], *alpha 2 macroglobulin* (*A2M*) [19], *secreted phosphoprotein 1 (SPP1)* [20], *pleiotrophin (PTN)* [21], several cell cycle genes [22] and *beta-defensin 11 (AvBD-11)* [23] in the chicken oviduct are detected predominantly in glandular epithelial cells of ovaries from laying hens with ovarian adenocarcinoma. Furthermore, there are several reports that over-expression of WNT4 is induced by its mutated regulator genes such as beta-catenin and GSK3B or aberrant expression of miRNAs in various cancer types [24], [25], [26]. Therefore, we hypothesized that expression patterns for WNT4 would differ between normal and cancerous ovarian tissues from laying hens and then determined whether cell-specific WNT4 expression was detectable in ovaries of laying hens with ovarian cancer. As illustrated in Figure 4A, quantitative PCR revealed that *WNT4* mRNA increased 3.35-fold (*P*<0.05) in cancerous ovaries as compared with normal ovaries of laying hens. Further, *WNT4* mRNA was localized predominantly to GE of cancerous ovaries, but not in any other cells including stroma and blood vessel (Figure 4B). However, *WNT4* mRNA was not detected in normal ovaries.

## Discussion

In the present study, we demonstrated that the *WNT4* gene is expressed in the isthmus and shell gland of the chicken oviduct in response to estrogen. In addition, expression of WNT4 is post-transcriptionally regulated by direct binding of its specific microRNA (*miR-1786*). Moreover, we found increased expression of *WNT4* in cancerous ovaries of laying hens that increased with age. These results support our hypothesis that WNT4 affects growth, differentiation and development of the chicken oviduct, and provide novel insights and concepts for further study of WNT4-related physiological processes in the laying cycle of hens and in ovarian carcinogenesis.

Among the WNT family members, WNT4 is a growth factor involved in development of a number of organs such as kidney, mammary gland and adrenal gland, as well as development of the reproductive tract in mammals [4]. In mice, *Wnt4* is highly expressed in the female reproductive tract with different expression patterns depending on stage of the estrous cycle and it is also involved in stromal cell proliferation and differentiation in the uterus which is required for implantation and decidualization during early pregnancy [5]. In addition, *Wnt4^−/−^* mice have an abnormal phenotype with respect to postnatal uterine development which renders female mice subfertile due to defects in implantation of blastocyst and subsequent survival, differentiation, and responsiveness to progesterone signaling by uterine stromal cells [5], [6]. However, WNT4 expression and function in the reproductive tract female chickens has not been reported previously.

Recently, Nicol and colleagues reported that WNT4 protein was highly conserved throughout vertebrate evolution [27]. The results also revealed that chicken WNT4 is likely to have similar physiological functions as for other vertebrate species. In the present study, we demonstrated expression of WNT4 in glandular (GE) and luminal epithelia (LE) of the isthmus and shell gland of the chicken oviduct (Figure 1). During passage of the egg through the oviduct, several layers of egg shell membranes that surround the yolk and the white are added sequentially as the egg mass passes through successive sections of the oviduct [28]. About 2 to 3 h after ovulation, the fertilized egg, with secretion of egg-white proteins including albumen from the magnum, passes into the isthmus which secretes various components of the soft shell membranes such as keratin-like protein, and types I, V and X collagens [29], [30]. The formation of the egg shell involving calcium deposition (approximately 5 to 6 g of calcium carbonate) is completed in the shell gland of the oviduct within 17 to 20 h after ovulation [28]. These results indicate that WNT4 may have roles in the oviductal epithelial cells of the chicken during egg formation and oviposition.

Results of the present study also demonstrated that DES stimulates WNT4 expression in GE of the isthmus and shell gland of the oviduct of developing chick reproductive tract (Figure 2). Indeed, DES induces massive growth of the juvenile oviduct and induces cytodifferentiation of epithelial cells into tubular gland cells, goblet cells, and ciliated cells [1]. In mice, expression of the *Wnt4* gene is regulated by estrogen during development of the uterus [5], [6]. There are changes in gene expression in the chick oviduct after exposure to DES [9] as DES binds strongly to estrogen receptor alpha (ESR1) to act as an agonist with effects similar to those induced by 17β-estradiol [1]. Thus, our results indicate that DES increases expression of WNT4 in a tissue- and cell-specific manner that is coordinate with development, differentiation and function of the various anatomical components of the chicken oviduct.

MicroRNAs (miRs) are short RNA molecules that control expression of certain genes by regulating mRNA stability and translation [31]. In chickens, hundreds of miRs have been identified, but functions are known for only a few miRs. In this study, we performed a miR target validation assay to determine whether specific miRs bind to the 3′-UTR of *WNT4* gene with the potential to affect transcription. As illustrated in Figure 3, co-transfection of eGFP-*WNT4* 3′-UTR and DsRed-*miR-1786* decreased the ratio of GFP-positive cells and GFP fluorescence density when compared to untreated control cells. Moreover, as shown in Figure 3E, *miR-1786* expression decreased *in vivo* in response to DES as compared to expression in oviducts of control chicks. These result revealed that WNT4 expression was influenced *in vitro* via its 3′-UTR which binds *miR-1786* directly and also *in vivo* wherein DES-mediated a decrease in *miR-1786*. Consequently, we found that *miR-1786* inhibits expression of *WNT4* in laying hens by regulating various post-transcriptional events that likely affect cellular processes in development, differentiation and oncogenesis in the reproductive tract of laying hens. However, further study is required to better understand its regulatory mechanism.

Ovarian cancer is a lethal gynecological malignancy and the overall 5-year survival rate from this disease is only 30 to 40% because most cases of the ovarian cancer are not detected at an early stage which makes it difficult to apply any type of therapy [32]. To overcome this problem, various animal models have been developed, but they have not proven to be as useful as the laying hen. However, the laying hen is recognized as the most appropriate animal model because they spontaneously develops ovarian cancer of the surface epithelium at a high rate as they age, as also occurs in women [11]. Indeed, *CTSB* [17], *SERPINB11* [15], *A2M* [19], and *PTN* [21] are genes that we reported to be most abundant in the GE of ovarian cancers in laying hens. In the present study, we found that WNT4 is highly expressed in the GE of cancerous as compared with normal ovaries of laying hens. These results strongly support the idea that the WNT4 expression is associated with initiation and development of human ovarian cancer and even more so as the individual ages [33]. Therefore, we suggest that WNT4 is likely to be involved in development of ovarian cancer as laying hen undergo aging.

In summary, results of this study demonstrated that *WNT4* is an estrogen-regulated gene during growth, development and differentiation of the chicken oviduct and that is likely plays a critical role in abnormal growth and function of cancerous ovarian cells of laying hens. These results provide new insights into the roles of WNT4 with respect to its hormonal regulation and post-transcriptional regulation of its expression in the oviduct and in ovarian tumors of laying hen.

## Materials and Methods

### Experimental Animals and Animal Care

The experimental use of chickens for this study was approved by the Institute of Laboratory Animal Resources, Seoul National University (SNU-070823-5). White Leghorn (WL) laying hens were subjected to standard management practices at the University Animal Farm, Seoul National University, Korea. All hens were exposed to a light regimen of 15 h light and 9 h dark with *ad libitum* access to feed and water.

### Tissue Samples

#### Study One

Following euthanasia of mature WL hens, tissue samples were collected from oviduct of 1- to 2- year-old females (n = 5). The collected samples were either frozen or fixed in 4% paraformaldehyde for further analyses. Frozen tissue samples were cut into 5- to 7-mm pieces, frozen in liquid nitrogen vapor, and stored at −80°C. The other samples were cut into 10 mm pieces and fixed in fresh 4% paraformaldehyde in PBS (pH 7.4). After 24 h, fixed tissues were changed to 70% ethanol for 24 h and then dehydrated and embedded in Paraplast-Plus (Leica Microsystems, Wetzlar, Germany). Paraffin-embedded tissues were sectioned at 5 mm.

#### Study Two

Female chicks were identified by PCR analysis using W chromosome-specific primer sets [34]. Treatment with DES and recovery of the oviduct (n = 5) were conducted as reported previously [10]. Briefly, a 15 mg DES pellet was implanted subcutaneously in the abdominal region of 1-week-old female chicks for 10 days. The DES pellet was removed for 10 days, and then a 30 mg dose of DES was administered for 10 additional days. Five 37-day-old chicks in each group were euthanized using 60%–70% carbon dioxide. The collected samples were either frozen or fixed in 4% paraformaldehyde for further analyses. Paraffin-embedded tissues were sectioned at 5 μm.

#### Study Three

Hens (n = 5 per time point) were euthanized at either 3 h or 20 h after ovulation using 60%–70% carbon dioxide. Samples of the magnum and the shell gland of oviducts from each hen were collected at each time point. Sampling of magnum and shell gland was at the middle of each tissue to prevent mixing with another tissue such as the infundibulum and isthmus. The tissue samples of similar size were: 1) removed and placed in Optimal Cutting Temperature (OCT) compound (Miles, Oneonta, NY); 2) frozen in liquid nitrogen and stored at −80°C; 3) fixed in freshly prepared 4% paraformaldehyde in PBS (pH 7.4); or 4) frozen immediately in liquid nitrogen and stored at −80°C until analyzed. After 24 h, tissues fixed in 4% paraformaldehyde were changed to 70% ethanol for 24 h and then dehydrated and embedded in Paraplast-Plus (Leica Microsystems, Wetzlar, Germany).

#### Study Four

A total 136 chickens (88 chickens aged over 36 months and 48 chickens aged over 24 months), which had completely stopped laying eggs were euthanized for biopsy and cancerous (n = 10) ovaries were collected. As a control, normal (n = 5) ovaries were also collected from egg-laying hens. We examined the tumor stage in 10 chickens with cancerous ovaries using characteristic features of chicken ovarian cancer [15], [35]. Three hens had stage III disease as ovarian tumor cells had metastasized to the gastrointestinal (GI) tract and liver surface with profuse ascites in the abdominal cavity. Five hens had tumor cells spread to distant organs including liver parenchyma, lung, GI tract and oviduct with profuse ascites, indicating stage IV disease. Two hens had stage I disease as tumors were limited to their ovaries. The collected samples were fixed in 4% paraformaldehyde for further analyses. After 24 h, fixed tissues were changed to 70% ethanol for 24 h and then dehydrated and embedded in Paraplast-Plus (Leica Microsystems, Wetzlar, Germany). Paraffin-embedded tissues were sectioned at 5 µm and stained with hematoxylin and eosin. Epithelial ovarian cancers in chickens were classified based on the cellular subtypes and patterns of cellular differentiation with reference to ovarian malignant tumor types in humans [35].

### RNA Isolation

Total cellular RNA was isolated from frozen tissues using Trizol reagent (Invitrogen, Carlsbad, CA) according to manufacturer's recommendations. The quantity and quality of total RNA was determined by spectrometry and denaturing agarose gel electrophoresis, respectively.

### RT-PCR Analysis

The expression of *WNT4* mRNA in chicken organs including the oviduct, ovary and cancerous ovary was assessed using RT-PCR as described previously [36]. The cDNA was synthesized from total cellular RNA (2 ug) using random hexamer (Invitrogen, Carlsbad, CA) and oligo (dT) primers and AccuPowerH RT PreMix (Bioneer, Daejeon, Korea). The cDNA was diluted (1:10) in sterile water before use in PCR. For *WNT4*, the sense primer (5′- GGA GTG CCA GTA CCA ATT CC -3′) and antisense primer (5′- CGT CGA ATT TCT CCT TCA GC -3′) amplified a 491-bp product. For *ACTB* (housekeeping gene), the sense primer (5′- GGC TGT GCT GTC CCT GTA TG -3′) and antisense primer primer (5′- ACC CAA GAA AGA TGG CTG GA -3′) amplified a 394-bp product. For *Ribosomal protein 4 (RPL4)* (housekeeping gene), the sense primer (5′- GGT ACT GGG AGA GCT GTT GC -3′) and antisense primer primer (5′- CCG GAA AGC TCT AAT GAT GC -3′) amplified a 465-bp product. The primers, PCR amplification and verification of their sequences were conducted as described previously [36]. PCR amplification was conducted using approximately 60 ng cDNA as follows: (1) 95°C for 3 min; (2) 95°C for 20 sec, 60°C for 40 sec and 72°C for 1 min for 35 cycles; and (3) 72°C for 10 min. After PCR, equal amounts of reaction product were analyzed using a 1% agarose gel, and PCR products were visualized using ethidium bromide staining. The amount of DNA present was quantified by measuring the intensity of light emitted from correctly sized bands under ultraviolet light using a Gel Doc^TM^ XR+ system with Image LabTM software (Bio-Rad).

### Quantitative RT-PCR Analysis

Total RNA was extracted from each segment of the oviduct and the ovary using TRIzol (Invitrogen) and purified using an RNeasy Mini Kit (Qiagen). Complementary DNA was synthesized using a Superscript® III First-Strand Synthesis System (Invitrogen). Gene expression levels were measured using SYBR® Green (Biotium, Hayward, CA, USA) and a StepOnePlus^™^ Real-Time PCR System (Applied Biosystems, Foster City, CA, USA). The *ACTB* and *RLP4* genes were analyzed simultaneously as reporter genes and used for normalization of data. These experiments were performed in triplicate. For *WNT4*, the sense primer (5′- GGA GTG CCA GTA CCA ATT CC -3′) and antisense primer (5′- AGA GAT GGC GTA GAC GAA CG -3′) amplified a 121-bp product. For *ACTB*, the sense primer (5′- CCC ATC TAT GAA GGC TAC GC -3′) and antisense primer primer (5′- CAC GCA CAA TTT CTC TCT CG -3′) amplified a 142-bp product. For *RLP4*, the sense primer (5′- GAA GAT TCA CCG CAG AGT CC -3′) and antisense primer primer (5′- GTT TTT GAT TCT GGG CAT GG -3′) amplified a 125-bp product. The PCR conditions were 94°C for 3 min, followed by 40 cycles at 94°C for 20 sec, 60°C for 40 sec, and 72°C for 1 min using a melting curve program (increasing the temperature from 55°C to 95°C at 0.5°C per 10 sec) and continuous fluorescence measurement. ROX dye (Invitrogen) was used as a negative control for the fluorescence measurements. Sequence-specific products were identified by generating a melting curve in which the C_T_ value represented the cycle number at which a fluorescent signal was significantly greater than background, and relative gene expression was quantified using the 2^–ΔΔCT^ method [37]. The 2^–ΔΔCT^ method is also known as the comparative C_T_ method. WNT4 expression was calculated using the following equation: ΔΔC_T_  =  ΔC_T, WNT4_ - C_T, reference gene._ These C_T_ value was normalized to the endogenous reference genes. For comparing *WNT4* expression between untreated and DES-treated oviducts in chickens, the relative quantification of gene expression was normalized to the C_T_ value of the untreated oviduct.

### *In Situ* Hybridization Analysis

Location of *WNT4* mRNA in sections (5 µm) of chicken oviducts and ovaries was determined by *in situ* hybridization analysis as described previously [21]. Briefly, for hybridization probes, PCR products were generated from cDNA primers used for RT-PCR analysis. The products were gel-extracted and cloned into pGEM-T vector (Promega). After verification of the sequences, plasmids containing gene sequences were amplified with T7- and SP6-specific primers (T7:5′-TGT AAT ACG ACT CAC TAT AGG G-3′; SP6:5′-CTA TTT AGG TGA CAC TAT AGA AT-3′). Then digoxigenin (DIG)-labeled RNA probes were transcribed using a DIG RNA labeling kit (Roche Applied Science, Indianapolis, IN). Tissues were collected, fixed in 4% paraformaldehyde, embedded in paraffin, sectioned at 5 μm and sections placed on APES-treated (silanized) slides. The sections were then deparaffinized in xylene and rehydrated to diethylpyrocarbonate (DEPC)-treated water through a graded series of alcohol. The sections were treated with 1% Triton X-100 in PBS for 20 min and washed twice in DEPC-treated PBS. The sections were then digested in 5 μg/ml Proteinase K (Sigma) in TE buffer (100 mM Tris-HCl, 50 mM EDTA, pH 8.0) at 37°C. After post-fixation in 4% paraformaldehyde, sections were incubated twice for 5 min each in DEPC-treated PBS and incubated in TEA buffer [0.1M triethanolamine containing 0.25% (v/v) acetic anhydride]. The sections were incubated in a prehybridization mixture containing 50% formamide and 4X standard saline citrate (SSC) for at least 10 min at room temperature. After prehybridization, the sections were incubated with a hybridization mixture containing 40% formamide, 4X SSC, 10% dextran sulfate sodium salt, 10 mM DTT, 1 mg/ml yeast tRNA, 1mg/ml salmon sperm DNA, 0.02% Ficoll, 0.02% polyvinylpyrrolidone, 0.2mg/ml RNase-free bovine serum albumin and denatured DIG-labeled cRNA probe overnight at 42°C in a humidified chamber. After hybridization, sections were washed for 15 min in 2X SSC at 37°C, 15 min in 1X SSC at 37°C, 30 min in NTE buffer (10 mM Tris, 500 mM NaCl and 1mM EDTA) at 37°C and 30 min in 0.1X SSC at 37°C. After blocking with 2% normal sheep serum (Santa Cruz Biotechnology, Inc., Santa Cruz, CA), the sections were incubated overnight with sheep anti-DIG antibody conjugated to alkaline phosphatase (Roche, Indianapolis, IN). The signal was visualized by exposure to a solution containing 0.4 mM 5-bromo-4-chloro-3-indolyl phosphate, 0.4 mM nitroblue tetrazolium, and 2 mM levamisole (Sigma Chemical Co., St. Louis, MO).

### MicroRNA Target Validation Assay

The 3′-UTR of *WNT4* was subcloned into pcDNA3eGFP (Clontech, Mountain View, CA) to generate the eGFP-miRNA target 3′-UTR fusion construct. For the dual fluorescence reporter assay, the fusion constructs containing the *DsRed* gene and *miR-1786,* were designed to be co-expressed under control of the CMV promoter. Both constructs were co-transfected into 293FT cells using the calcium phosphate method. When the DsRed-miRNA is expressed and binds to the target site of the 3′-UTR downstream of the GFP transcript, green fluorescence intensity decreases due to degradation of the GFP transcript. At 48 h post-transfection, dual fluorescence was detected by fluorescence microscopy and calculated by FACSCalibur flow cytometry (BD Biosciences). For flow cytometry, the cells were fixed in 4% paraformaldehyde and analyzed using FlowJo software (Tree Star Inc., Ashland, OR).

### Statistical Analyses

All quantitative data were subjected to analysis of variance (ANOVA) according to the general linear model (PROC-GLM) of the SAS program (SAS Institute, Cary, NC). All tests of significance were performed using the appropriate error terms according to the expectation of the mean square for error. Data are presented as mean ± SEM unless otherwise stated. Differences in the variance between untreated and DES-treated oviducts were analyzed using the F test, and differences in the means were subjected to Student's t test. Differences were considered significant at *P*<0.05.
